# Lipidation as a post-translational code for protein liquid-liquid phase separation

**DOI:** 10.1016/j.jlr.2026.101001

**Published:** 2026-02-13

**Authors:** Soodabeh Abbasi Sani, Agnieszka Chytła, Martin Sztacho

**Affiliations:** Laboratory of Cancer Cell Architecture, Institute of Biochemistry and Experimental Oncology, First Faculty of Medicine, Charles University, Prague, Czech Republic

**Keywords:** phosphoinositides, post-translational modifications, biomolecular condensates, membrane organization, lipid rafts, transcription, phospholipids, cell signaling

## Abstract

Liquid–liquid phase separation has emerged as a central organizing mechanism that drives the formation of biomolecular condensates and enables cells to spatially and temporally coordinate metabolism, signaling, and gene expression. While the influence of post-translational modifications such as phosphorylation and ubiquitination on condensate behavior is well-established, the contribution of lipidation, the covalent attachment of lipid moieties to proteins, to these processes has received far less attention. Lipidation dictates protein hydrophobicity, membrane affinity, and subcellular distribution, yet how these parameters influence LLPS and thereby modulate condensate dynamics remains unclear. We propose that lipidation operates as a molecular code that integrates membrane association with phase separation, thereby tuning the assembly, composition, and thus functional output of condensates. Extending this concept beyond classical membrane systems, we further suggest that nuclear phosphoinositides, particularly phosphatidylinositol 4,5-bisphosphate (PI(4,5)P2), may act as an unconventional lipid modifier that structures membraneless nuclear compartments through a process termed PIPoylation. Drawing on recent findings, we outline how canonical covalent lipidations, including palmitoylation, myristoylation, prenylation, and phospholipidation, govern membrane nanodomain organization, autophagy, and nuclear condensate architecture. We discuss how covalent lipidation influences condensate wetting, membrane curvature, and lipid–protein demixing, and how PI(4,5)P2 metabolism links chromatin remodeling with transcriptional control via LLPS. Together, these mechanisms underscore lipidation as a crucial regulator of condensate-membrane communication across cellular compartments.

Inside the eukaryotic cell, finely tuned regulatory networks coordinate essential processes such as signal transduction, stress responses, and protein homeostasis, while enabling rapid adaptation to environmental changes (1, 2, 3, 4, 5). One fundamental mechanism underlying this regulation is liquid-liquid phase separation (LLPS), which allows cells to compartmentalize biochemical reactions either within membrane-bound organelles or by forming membraneless structures known as biomolecular condensates (BMCs) (6). These dynamic compartments arise through weak, multivalent interactions among nucleic acids, lipids and proteins with multiple interaction domains, intrinsically disordered regions (IDRs) and/or RNA-binding domains (RBDs). Such interactions enable phase separation and contribute to spatial and temporal organization within cells (7). In addition to these established interactions, lipids and lipid-rich microenvironments have been increasingly recognized as modulators of condensate formation and stability, functioning by altering local physicochemical properties and providing additional interaction platforms for phase-separating components (8). These multivalent interactions not only drive condensate formation but also contribute to how molecules distribute between phases. In particular, the patterning of charged residues within IDRs has been shown to influence selective partitioning within BMCs (9). BMCs are present in both the cytoplasm and the nucleus, and their composition and molecular architecture vary across cell types and physiological states (10, 11). Protein post-translational modifications (PTMs) are central regulators of BMC formation, maintenance, and dissolution. Together with protein physicochemical properties and parameters such as component concentration, charge, pH, and temperature, PTMs modulate the molecular interactions that govern condensate dynamics (7, 12). PTMs are covalent enzymatic modifications of protein side chains that influence protein function, structure, localization, stability, and degradation, thereby shaping their cellular fate. Modifications such as phosphorylation, acetylation, and ubiquitination have been shown to regulate BMC assembly-disassembly cycles (12, 13). Conversely, PTM dysregulation disrupts condensate dynamics and is associated with the progression of numerous diseases, including cancer and neurodegenerative disorders such as Alzheimer, Parkinson, and Huntington disease (7, 11, 14, 15, 16). In cancer, LLPS contributes to the dynamic organization of signaling complexes that regulate cell migration, and PTMs fine-tune this process by controlling transitions between distinct motility states during metastasis (17, 18, 19, 20). Collectively, current research highlights the key role of PTMs in regulating phase separation and their implications for cellular physiology and disease.

While most studies have focused on phosphorylation and ubiquitination as key regulators of LLPS in BMCs, another important class of PTMs has received comparatively little attention. Lipidation, defined as the addition of a lipid moiety to the protein, has been used in literature to describe both non-covalent lipid-protein associations and covalent lipid modifications. These interactions can influence protein stability, conformation, and activity as well as their association with cellular membranes and specific subcellular compartments (21, 22, 23, 24). Importantly, these two forms of lipidation differ fundamentally in their mechanisms of action. Non-covalent lipid association, exemplified by apolipoprotein A1, relies on hydrophobic interactions that enable lipid binding without chemical modification of the protein backbone (24). By contrast, covalent lipidation refers to enzymatically catalyzed post-translational modifications in which lipid moieties are stably attached to specific amino acid residues, including myristoylation, palmitoylation, prenylation, glycosylphosphatidylinositol (GPI) anchoring, and phospholipidation. Through these distinct mechanisms, lipidation can modulate the spatial distribution, structural dynamics, and functional properties of phase-separating proteins, particularly under stress conditions or in disease contexts (25).

This review primarily addresses covalent lipidation as a regulatory post-translational modification influencing membrane-associated protein organization, protein conformation, and the formation of membraneless condensates. Particular emphasis is placed on nuclear PI(4,5)P2 as a potentially unconventional covalent form of protein lipidation, as suggested by emerging evidence.

## Membrane-driven Protein relocalization and nanodomain organization

**Fig. 1:**
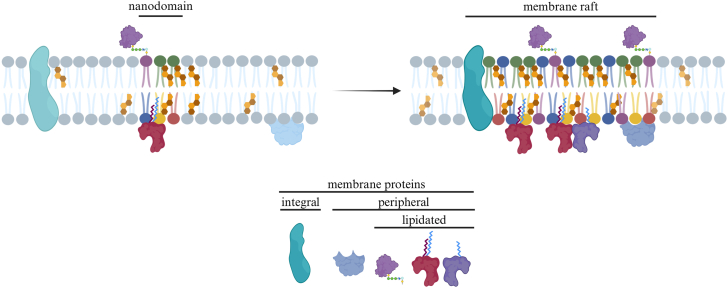
Coalescence of lipid nanodomains into stabilized membrane rafts/Membrane rafts formation. The plasma membrane contains small, dynamic nanodomains enriched in sphingomyelin, cholesterol, and proteins (left). These can coalesce into larger, more stable membrane rafts (right), which act as organizational platforms for signaling and membrane trafficking. Created in https://BioRender.com.

The plasma membrane is far more than a simple barrier enclosing the cell. It is a dynamic, highly organized platform involved in a variety of cellular processes from signal transduction to cell adhesion (26). This two-dimensional structure is composed of a mosaic of lipids and proteins, asymmetrically distributed across the bilayer (27, 28). Its integrity is maintained by hydrophobic interactions among the acyl chains of saturated and unsaturated lipids, which drive bilayer formation and determine membrane packing and fluidity. Cholesterol plays a pivotal role by inserting between the acyl tails of other lipids, reducing permeability and modulating fluidity according to the local lipid composition (29). These lipid-lipid interactions enable the membrane to self-organize laterally, giving rise to specialized domains, commonly referred to as membrane rafts (previously lipid rafts) (30). Such domains form, at least partially, through LLPS, as distinct lipid–lipid interactions produce local differences in lateral mobility across the bilayer (31). Small, dynamic nanodomains enriched in sphingomyelin, cholesterol, and proteins can merge to form more stable domains (Fig. 1). These, in turn, may coalesce into even larger (>200 nm) platforms that enable the precise regulation of a wide range of cellular processes, including signaling, membrane trafficking or viral entry (32, 33, 34, 35, 36). Accumulating evidence indicates that membrane nanodomains do not arise solely from lipid–lipid interactions but instead involve the interplay between lipid composition, membrane protein clustering and oligomerization, and actin cytoskeleton (32, 37). Although these factors can promote domain formation in model membranes and substantial progress has been made in defining the underlying physicochemical principles, the molecular mechanisms governing membrane raft formation and dynamics in living cells remain elusive (32, 37). However, within this framework, lipid-protein interactions are thought to play a central role in stabilizing these nanodomains, and many of these interactions depend on lipidation (38, 39).

The selective recruitment of proteins into membrane rafts is mediated by distinct modes of protein–membrane interaction. Membrane proteins can be classified as integral or peripheral, the latter associating through lipid-binding motifs, electrostatic interactions, clusters of charged residues, or lipidations (21, 40). Beyond acting as membrane-targeting signals, lipidation also alters the physicochemical properties of proteins by increasing their lipophilicity (21, 41, 42). In this context, lipophilicity reflects the additive contribution of attached lipid moieties to the hydrophobic free energy of the protein, which determines the strength of protein association with membranes (43, 44, 45, 46). The molecular features of the lipid moieties that govern this behavior include chain length, saturation, and multiplicity (45, 47, 48). Even modest increases in acyl chain length can have measurable effects, with each added methylene group contributing on the order of ∼0.8 kcal/mol to hydrophobic free energy and thereby strengthening protein association with lipid membranes (49, 50, 51). Longer and more saturated acyl chains also tend to pack more tightly within the membrane and reduce local hydration, further stabilizing protein-membrane interactions (52). This is well illustrated by H-Ras and N-Ras proteins, where prenylation provides an initial membrane anchor that is further stabilized by reversible palmitoylation (53). A similar mechanism applies to flotillin-2, which undergoes irreversible N-myristoylation complemented by reversible S-palmitoylation at multiple cysteine residues. This combination contributes to flotillin-2 localization within membrane nanodomains (54). In addition, differences in lipid saturation can influence protein interactions towards regions of the membrane with distinct packing or fluidity, without necessarily affecting the overall affinity for the membrane (55, 56). Beyond individual protein-membrane interactions, covalently lipidated proteins can promote lateral organization within the membrane, facilitating the formation and stabilization of functional nanodomains. A notable example is postsynaptic density protein 95 (PSD95), whose palmitoylation has been implicated in the nucleation of membrane rafts in neurons (57). Similarly, another palmitoylated protein, membrane palmitoylated protein 1 (MPP1), has been shown to participate directly in the organization and stabilization of flotillin-based raft nanodomains, both in living cells and in artificial membrane models (58, 59). Other lipid modifications also play critical roles in membrane domain organization. For instance, myristoylation of Src family kinases, such as Fyn and Lck, is required for their localization to membrane rafts, where they play a pivotal role in T cell receptor activation, highlighting the importance of membrane rafts in orchestrating immune signaling (60). During HIV-1 infection, the myristoylated Gag protein associates with PI(4,5)P2 at the plasma membrane, promoting viral assembly and the formation and/or stabilization of membrane rafts to facilitate efficient virus budding (61, 62, 63). Since membrane rafts act as the main regulators of signal transduction, they are often affected during the progression of disease (64, 65, 66). Both the disruption and the formation of membrane rafts have been shown to contribute to cancer metastatic processes, including angiogenesis, epithelial-to-mesenchymal transition, migration, and adhesion (67, 68, 69, 70, 71, 72, 73, 74). A key regulator of cell migration, CD44 localizes to membrane rafts through palmitoylation. This localization restricts its interactions with migratory partners such as Ezrin. However, studies on breast cancer cells have shown reduced levels of CD44 palmitoylation, accompanied by its relocation out of membrane rafts to less ordered parts of the membrane. This relocation enables CD44 to interact with ezrin, thereby driving actin remodeling and, consequently, increasing migration, which contributes to a more aggressive cellular phenotype (71, 72, 73).

## Membrane-induced protein phase separation and condensate dynamics

Actin remodeling is regulated through both membrane raft organization at the plasma membrane and LLPS-driven BMCs that assemble key actin regulators. Multivalent interactions among neuronal Wiskott-Aldrich syndrome protein (N-WASP), Nck, and phosphorylated nephrin drive the assembly of BMC, which in turn enhances actin polymerization mediated by Arp2/3. Notably, nephrin, a transmembrane protein, tethers these condensates to the plasma membrane, providing an example of how membrane-associated proteins can physically couple condensates to the cell surface (75, 76, 77, 78).

BMCs attach to membranes using the same physical principles that control how droplets interact with surfaces. The degree of contact depends on the balance of forces at the interface (79). In cells, this balance is influenced by lipid packing, protein lipophilicity, local charge, and protein-lipid interactions (80). As a consequence, condensates tend to associate with membrane regions whose physical properties support stable contact. Much like liquid droplets on structured surfaces, BMCs behave as deformable liquid phases. When immiscible droplets approach one another, their adhesion, bridge formation, and coalescence are governed by the interplay of interfacial tensions, and on nanostructured surfaces, these interactions can trigger wetting transitions that alter droplet spreading (79, 81).

**Fig. 2:**
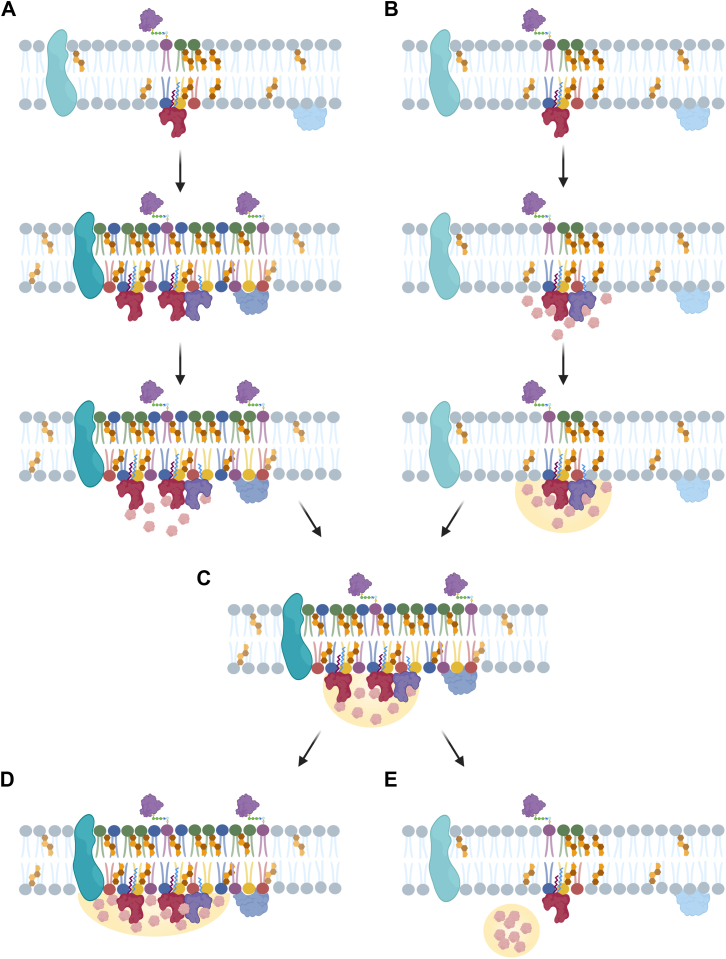
Reciprocal coupling between membrane organization and condensate formation. A: Membrane organization and raft formation can promote LLPS and condensate formation. B: Conversely, condensate adhesion can drive membrane reorganization. Both mechanisms lead to (C) partial condensate wetting of the membrane, followed by either (D) complete spreading along the membrane or (E) dewetting, where condensate detaches and no longer interacts with the membrane. Created in https://BioRender.com.

Similar principles apply at cellular membranes, where the extent of condensate-membrane contact influences condensate stability, spreading, and coalescence. A key determinant of condensate-membrane association is the lipid order of the bilayer, with disordered membranes promoting stronger condensate wetting at the membrane interface (82, 83). The degree of membrane wetting can range from partial contact (Fig. 2C) to complete spreading (Fig. 2D) or dewetting (Fig. 2E), in which condensates remain fully separated from the membrane. The extent of wetting is governed by the balance of interfacial tensions and surface energies. These interactions can influence downstream processes, including actin remodeling and signal transduction. (84, 85, 86).

Beyond membrane-associated regulation, condensate behavior is also modulated by PTMs. Acting together with parameters such as concentration, charge, pH, and temperature, PTMs modulate the molecular interactions that govern selective partitioning into condensates, their formation, maintenance, and dissolution, collectively known as condensate dynamics (12, 83, 87, 88). This type of regulation has been described for multiple condensates, including nephrin-Nck-N-WASP and the linker for activation of T cell (LAT) condensates, where phosphorylation controls assembly and disassembly at the plasma membrane (76, 89). Although phosphorylation, ubiquitination, acetylation and methylation have received the majority of attention in the context of LLPS, less-studied PTMs also contribute to LLPS and BMC formation (22, 90, 91). Among them, lipidation stands out, as it alters protein properties closely linked to condensate formation, particularly lipophilicity and membrane association.

**Fig. 3:**
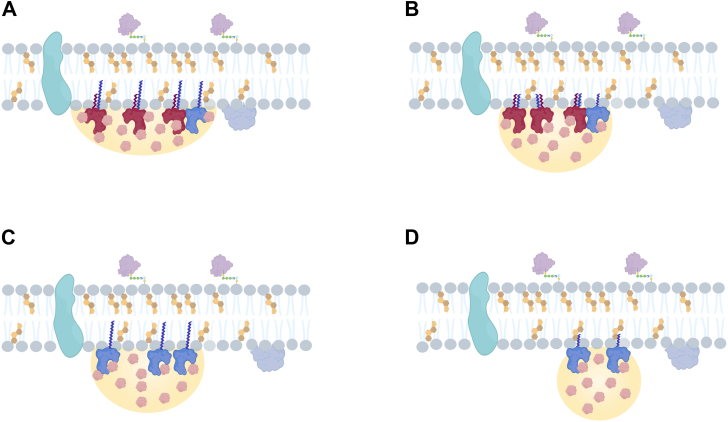
Lipidation-dependent modulation of condensate dewetting. Changes in protein lipophilicity introduced by covalent lipidation may modulate condensate association with lipid membranes. A: Multiple lipidations with longer acyl chains are expected to markedly increase protein lipophilicity, thereby lowering the energetic cost of membrane-associated condensation and stabilizing condensate–membrane contact. By contrast, (B) multiple lipidations with shorter acyl chains or (C) single lipid modification with longer acyl chains are expected to support only transient stabilization of condensate-membrane interactions. D: Single lipid modifications with a short acyl chain are predicted to generate the lowest increase in lipophilicity, resulting in weak and limited condensate association with the membrane. Created in https://BioRender.com.

These lipidation-dependent changes in protein lipophilicity and membrane association are therefore expected to proportionally affect condensate formation and membrane association. Consistent with this, increased lipophilicity is expected to support more stable interactions between condensates and lipid interfaces (Fig. 3). Accordingly, multiple lipid attachments with longer acyl chains are expected to enhance protein lipophilicity, thereby reducing the free-energy cost of membrane association and promoting membrane-associated condensation with more stable and longer-lived membrane interactions (Fig. 3A). In contrast, multilipidations with shorter acyl chains (Fig. 3B), or single lipidation with a longer acyl chain (Fig. 3C), are expected to result in limited condensate spreading and thus transient membrane association. Proteins modified with a single short acyl chain lipid moiety are therefore expected to display minimal condensate spreading and limited retention at membrane interfaces (Fig. 3D). Consequently, even modest changes in protein lipophilicity may translate into pronounced differences in condensate-membrane interactions, reflected in altered wetting behavior, contact area, and spreading at lipid interfaces.

Condensate interactions can also remodel the membrane itself by inducing curvature, promoting invagination, and reorganizing lipid domains (82, 92). Interestingly, this relationship seems to be reciprocal, as lipid membranes can potentiate condensate formation, while BMCs, in turn, can promote membrane organization (Fig. 2) (82, 86, 92, 93). This interplay can be observed in T cell signaling, where T cell activation triggers the formation of BMC that associate with and stabilize membrane rafts. In turn, membrane rafts can nucleate and stabilize the LAT condensates, both in vitro and in living cells (86). Such interactions provide an additional layer of compartmentalization, allowing cells to fine-tune physiological processes. Thus, condensate-membrane interactions that emerge are manifestations of the same hydrophobic principles that govern protein self-assembly and membrane organization.

While the lipidation-driven protein-membrane interplay discussed above primarily involves the recruitment of proteins to pre-existing membrane domains, thereby increasing their local concentration and promoting condensate formation, selective autophagy represents a striking inversion of this principle. In this pathway, phase-separated cargo-receptor condensates form first and act as organizational hubs, recruiting the autophagy machinery and initiating phagophore biogenesis. As the membrane expands, microtubule-associated protein 1 light chain 3 (LC3) phospholipidation anchors key autophagy factors to the growing phagophore, thereby coupling membrane dynamics to condensate localization. This mechanism shows how cells can reverse the direction of membrane-condensate interactions, coordinating the recognition of cargo with the formation of autophagosomes with precision.

## Condensate-induced protein lipidation and membrane recruitment

Phospholipidation is a specific subtype of protein lipidation characterized by the covalent attachment of phospholipids to proteins. A well-characterized example of a phospholipidated protein is the LC3, a mammalian ortholog of the yeast Atg8 protein (94). LC3 is a key regulator of both canonical and non-canonical autophagy pathways. Its phospholipidation is catalyzed by ATG3, which mediates the conjugation of LC3 isoform, LC3-I, to the phosphatidylethanolamine (PE) headgroup on the phagophore, generating the LC3-II and promoting autophagy (95, 96). This modification is particularly critical during nutrient starvation, when canonical degradation pathways are insufficient (97). Moreover, the kinase activity of class III phosphatidylinositol 3-kinase complex I (PI3KC3-C1) and the presence of phosphatidylinositol 3-phosphate (PI3P) are essential for LC3 lipidation and successful autophagosome formation (98).

**Fig. 4:**
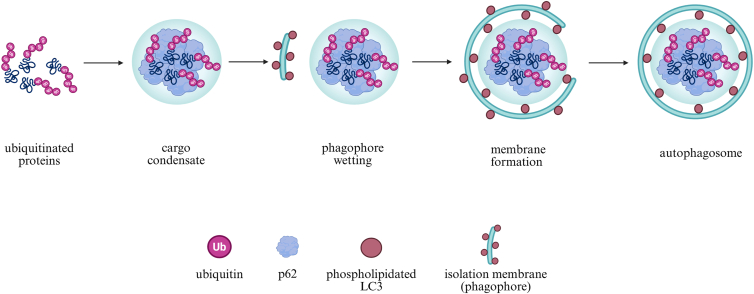
Selective-autophagy condensates direct autophagosome formation. Polyubiquitinated proteins recruit and concentrate p62, which undergoes LLPS to form a cargo condensate that defines the site of autophagosome initiation. The condensate then engages lipidated LC3 on the nascent phagophore, establishing stable membrane wetting and positioning the phagophore at the condensate surface. This interaction guides local membrane expansion and curvature, enabling the phagophore to progressively wrap around the condensate. Continued growth and closure of the membrane ultimately generate a mature autophagosome enclosing the p62-ubiquitin cargo. Created in https://BioRender.com.

While membrane nucleation typically precedes cargo capture in canonical autophagy, selective autophagy is noteworthy because it reverses this sequence. Phase-separated cargo-receptor condensates form first and guide the subsequent membrane wetting and encapsulation (99, 100). In selective autophagy, cargo and its receptors play a central role, acting as templates that initiate autophagosome formation rather than serving merely as passive targets for autophagy. Polyubiquitin chains, rather than the cargo itself, provide the signal that drives the formation of selective-autophagy condensates (101). Autophagy receptors bind these ubiquitin chains and simultaneously interact with phospholipidated LC3 through their LC3-interacting region (LIR) motifs, thereby enabling condensates to tether to the autophagosomal membrane (Fig. 4). p62 illustrates this dual function: it forms phase-separated condensates with polyubiquitin chains while acting as an LC3 adaptor during autophagosome biogenesis (85, 99, 100, 102). Phosphorylation of serines within the LIR region (S349, S365, S366, and S370) increases the negative charge of p62, enhancing its electrostatic attraction to positively charged domains, such as the FIP200 claw domain (100). This interaction forms a molecular bridge that recruits the ULK1 complex (ULK1-ATG13-FIP200-ATG101) to ubiquitinated condensates, leading to ULK1 activation and initiation of autophagy (103).

Phospholipidation then ensures that p62 condensates, previously stabilized and primed by phosphorylation for FIP200/ULK1 recruitment, are physically linked to the growing membrane (103). Phosphorylation, therefore, not only stabilizes p62 condensates and enhances phase separation but also concentrates the components required for membrane formation at the site of cargo assembly. Through these coordinated steps, the spatial organization of phase-separated condensates guides the wrapping of cargo by the growing membrane, integrating molecular cues with membrane dynamics to ensure precise envelopment and drive autophagosome biogenesis.

The concept of protein phospholipidation as a regulatory mechanism is not confined to cytoplasmic processes. Increasing evidence suggests that this modification may also occur within the nucleus, which is an environment largely devoid of membranes (except for the nuclear envelope) but enriched in membraneless condensates formed through phase separation.

## Nuclear phosphoinositides and their potential role in phospholipidation

Among various lipid species, phosphoinositides (PIPs) have emerged as key candidates for mediating dynamic roles in nuclear phospholipidation. These negatively charged, amphiphilic molecules comprise a hydrophobic acyl tail and a hydrophilic inositol head group, a structure that enables them to participate in diverse protein-lipid and lipid-lipid interactions (104, 105). While PIPs are best known for their role in cytoplasmic processes such as actin cytoskeleton remodeling, signal transduction, and cell proliferation, their presence within the nucleus was initially controversial (106, 107, 108, 109, 110, 111). The initial findings demonstrating the presence of phosphoinositides within the nucleus, independent of the nuclear envelope, date back nearly four decades (112, 113, 114). The first compelling evidence came from biochemical studies that used detergent-treated nuclei and radiolabeled fatty acid tracing to eliminate nuclear membranes and cytoplasmic contamination, thereby demonstrating that nuclear phospholipids could exist independently of membranes (112, 115). Subsequent studies demonstrated that membrane-free nuclei can synthesize PI(4,5)P2, indicating that nuclear phosphoinositides can be generated in the nucleus (112). These findings were further supported by the detection of radiolabeled phosphate incorporation into nuclear phosphoinositides, indicating active intranuclear lipid metabolism (116). Beyond demonstrating their existence and metabolism, later studies showed that nuclear phosphoinositides are spatially organized within the nucleus and that key enzymes involved in phosphoinositide metabolism are present within the nuclear compartments (105, 108, 110, 112, 117, 118). More than 40% of the nuclear PIP pool exists in a non-membrane-bound state, localized to distinct nuclear bodies such as nucleoli, nuclear speckles, dynamic, membraneless nuclear compartments enriched in RNA-processing and splicing factors and nuclear lipid islets (NLIs) (119, 120, 121).

**Fig. 5:**
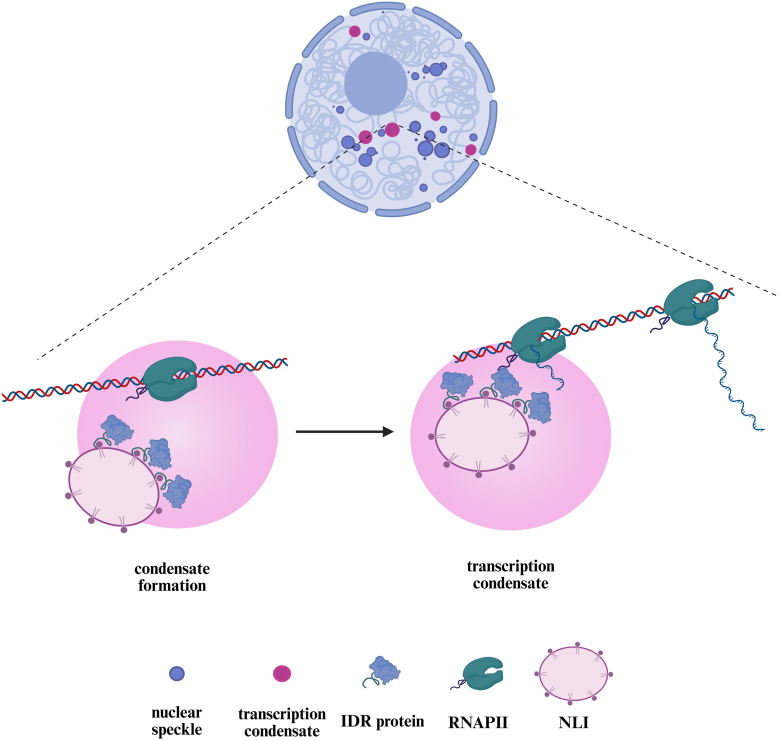
Nuclear lipid islets nucleate and organize transcription condensates. Nuclear lipid islets (NLIs) are membrane-less compartments enriched in PI(4,5)P2, RNA, and transcriptional regulators. By recruiting IDR-containing transcription factors and RNA polymerase II (RNAPII), NLIs promote LLPS and stabilize transcription condensates. RNAPII is responsible for initiating transcription within the condensate. Subsequently, RNAPII transitions to productive elongation, which entails its movement away from the condensate along the DNA template. Concurrently, the nascent RNA transcript extends outside the condensate. Created in https://BioRender.com.

NLIs were recently identified as a novel class of membrane-less nuclear compartments enriched in PI(4,5)P2, RNA, and proteins involved in transcriptional regulation (Fig. 5) (122). These structures act as spatial scaffolds that recruit factors such as nuclear myosin 1 (NM1) and the RNA polymerase II (RNAPII) complex. Through these interactions, NLIs stabilize transcriptional condensates and promote the organization of the transcription machinery, processes essential for restoring transcription in NM1-deficient cells (122). Although NLIs exhibit properties consistent with condensate-like behavior, direct evidence demonstrating LLPS as their assembly mechanism is still unknown. RNAPII transcription is regulated by phosphorylation of its C-terminal domain, with Ser5 phosphorylation marking initiation stage within nucleoplasmic condensates, whereas Ser2 phosphorylation is associated with transcription elongation and later stages of transcription in the proximal regions of nuclear speckles (123, 124, 125, 126, 127, 128). These stages are connected to specific phosphoinositides: PI(4,5)P2 enhances condensation of the RNAPII initiation complex, recruiting scaffold proteins such as bromodomain-containing protein 4 (BRD4), and thereby enhancing efficient transcription initiation. In contrast, PI(3,4)P2 is linked to elongation and co-cluster with nuclear speckle markers such as SON, suggesting a role in later stages of RNAPII transcription processing within these nuclear compartments (129, 130, 131, 132).

Within the nucleus, the most abundant and well-characterized species is PI(4,5)P2. It interacts with both proteins and RNA, influencing the assembly and organization of nuclear structures (105, 118, 133, 134, 135). Like other PIPs, PI(4,5)P2 can bind canonical PIP-binding domains such as pleckstrin homology (PH), Fab1-YOTB-Vac1-EEA1 (FYVE), Phox homology (PX), 4.1 protein-ezrin-radixin-moesin (FERM), and plant homeodomain (PHD) finger domains (136, 137, 138). However, proteomic studies have shown that in the nucleus, most PIP-binding proteins recognize PI(4,5)P2 through unstructured polybasic regions rich in lysine and arginine residues (K/R motifs) (119, 120). These sequences are commonly found in proteins involved in gene expression, RNA processing, and chromatin organization and are also characteristic of RNA-dependent PI(4,5)P2-associated nuclear proteins (139). By activating the BRG/BRM-associated factor (BAF) complex, PI(4,5)P2 promotes the assembly of dynamic chromatin hubs where transcriptional machinery and remodelers converge into transient condensates (140). This process illustrates how phosphoinositides can act as allosteric regulators and scaffolds that stabilize multivalent interactions, linking lipid signaling to condensate dynamics and nuclear architecture.

Emerging evidence suggests that specific patterns of altering blocks of charged residues within the protein IDRs contribute to selective nuclear compartmentalization (9). Disrupting or modifying this pattern can redirect proteins, altering their localization and functional output, while proteins with similar charge motifs tend to co-partition and carry out analogous roles (9). According to the active microemulsion model, multivalent interactions among IDRs and RNA promote LLPS, forming microdomains whose size and stability are constrained by amphiphilic molecules (141). As an amphiphilic lipid capable of modulating protein-RNA, protein-lipid, and protein-protein interactions, PI(4,5)P2 appears to be a strong candidate for coordinating nuclear architecture.

Recent studies suggest that the interaction between nuclear PI(4,5)P2 and transcriptional regulators, such as the BRD4 and mediator complex subunit 1 (MED1), both of which contain IDRs, is RNA-dependent (8, 139). RNA oligomerization and secondary structure appear to enhance the recruitment and spatial organization of PI(4,5)P2 within phase-separated nuclear compartments (8, 139). Moreover, PI(4,5)P2 binds AU-rich RNA motifs and the long non-coding RNA HANR, forming complexes that localize to the perinucleolar compartment (PNC) in proximity to oncogenic super-enhancers (142). Manipulating nuclear PI(4,5)P2 levels by silencing its biosynthetic enzyme PIP5Kα1 or its phosphatase SHIP2 does not affect HANR localization within the PNC but instead increases nucleoplasmic dispersion and overall RNA abundance. These findings suggest that PI(4,5)P2 plays a regulatory role in RNA distribution and stability within the nucleus (139, 142).

The ability of PI(4,5)P2 to perform complex regulatory functions within a membrane-less nuclear environment suggests the presence of additional mechanisms that enable it to remain organized and functional in the absence of a membrane scaffold. Experimental studies demonstrate that PI(4,5)P2 is present in structures such as nuclear speckles and nucleoli, where its headgroup remains exposed to the nucleoplasm (120). These observations raise important questions about the molecular basis of PI(4,5)P2 incorporation into nuclear condensates. One explanation proposes that PI(4,5)P2 integrates into the core of biocondensates, where it acts as an amphiphilic modulator (8, 141). Another possibility is that PI(4,5)P2 behaves as a typical amphiphile, self-assembling into organized aggregates such as micelles that shield its hydrophobic acyl chains from the surrounding nucleoplasm. It has also been suggested that similar to the nuclear receptor Steroidogenic Factor 1 (SF-1), certain PIP-interacting proteins may contain hydrophobic binding pockets that accommodate the lipid acyl chains while leaving the hydrophilic inositol head exposed for intermolecular interactions (143, 144). Finally, emerging evidence suggests that nuclear proteins might themselves become covalently linked to phosphoinositides, representing a novel form of phospholipidation (145). In this mechanism, the covalent attachment of a PIP would drive the protein into a more energetically favorable conformation in which the acyl chains are buried and thus shielded from the aqueous environment, while leaving the highly charged inositol headgroup accessible for interactions. Sztacho *et al.* showed that the motifs within nuclear proteins responsible for transient binding to PIPs are hydrophilic, suggesting that PIP-protein interactions occur primarily through the headgroup rather than through the hydrophobic acyl chains (120). These interactions could stabilize protein assemblies in membraneless nuclear compartments into biomolecular condensates. Moreover, the presence of the amphiphilic moiety as the covalent modification provides a conceptual connection to the active microemulsion model proposed by Hilbert *et al.*, where amphiphile limits the size of the condensate (141). Taken together, these possibilities suggest multiple strategies that PI(4,5)P2 may employ to maintain its organization in membraneless condensates, with phospholipidation representing a particularly intriguing example.

## Phosphoinositol code and the potential of novel PTM

Phosphoinositides function not only as dynamic signaling molecules but also as key regulators of vesicular trafficking and membrane lipid distribution by defining membrane identity through their differentially phosphorylated inositol headgroups (146, 147). In addition to these membrane-associated roles, the various phosphorylation patterns of nuclear phosphoinositide pools, together with their ability to shape local charge environments and influence multivalent interactions, position them as important modulators of nuclear condensates, chromatin architecture, and gene expression, potentially acting through a broader ‘phosphoinositol code’ (130). In this context, covalent modification of nuclear proteins through phospholipidation emerges as a potential mechanism by which phosphoinositides may interact directly with the molecular scaffolds of condensates. Such a mechanism could provide an additional layer of spatial and functional control over phase separation-driven processes, enabling phosphoinositides to act as preassembled components of membrane-less nuclear structures that fine-tune their biochemical environment and condensation dynamics.

The first insights into the possibility of PI(4,5)P2-phospholipidation, hereafter referred to as PIPoylation, came from studies of myelin basic protein (MBP), a structural component of the myelin sheath (148, 149, 150). MBP is a protein that contains an IDR domain, adopts a structure upon lipid association and predominantly localizes between the cytoplasmic leaflets of oligodendrocytes, where it forms an amorphous phase that mediates adhesive membrane interactions (151, 152). Early biochemical studies identified a lipopeptide in which a phospholipid moiety, most likely PI(4,5)P2, was covalently attached to Ser54 of MBP (148, 149). Further experiments using MBP depleted of endogenous phospholipids demonstrated that incubation with radiolabeled exogenous phospholipids generated the same lipopeptides as those previously observed for MBP isolated from radiolabeled myelin. Because the exogenous radiolabeled phospholipid preparation contained no detectable contaminants, these findings were interpreted as evidence that PIPoylation occurs through direct covalent attachment of the phospholipid to the protein rather than through ATP-dependent phosphorylation (150, 153). However, discrepancies remain regarding the nature of the bond, and the mechanism underlying PI(4,5)P2 conjugation remains unresolved.

More recent work has extended the concept of PIPoylation to nuclear biology and viral infection (145). PI(4,5)P2 influences multiple stages of the viral life cycle, including entry, plasma membrane assembly, and the intracellular transport and accumulation of viral components. Within the nucleus, it associates with both host and viral proteins in BMCs, modulating RNAPII transcription, RNA metabolism, and chromatin organization (154, 155). Human papillomaviruses (HPVs) are among the viruses known to exploit these mechanisms. In HPV8-associated skin cancer, the HPV8 E6 oncoprotein disrupts PI(4,5)P2 metabolism, leading to elevated nuclear PI(4,5)P2 levels and altered PIPoylation patterns across ten nuclear proteins (145). One of these, staphylococcal nuclease domain-containing protein 1 (SND1/p100), is a multifunctional RNA-binding protein that interacts with RNAPII and links the transcription factor STAT6 to RNAPII (156). Given that SND1 is overexpressed in several cancers, its PIPoylation may represent a mechanistic connection between dysregulated PI(4,5)P2 signaling and tumorigenesis (156, 157, 158, 159). Another identified protein was cullin associated and neddylation dissociated 1 (CAND1), a regulator of Cullin-RING ubiquitin ligases that acts as an F-box exchange factor (139, 145, 160). Elevated CAND1 expression has been associated with tumor aggressiveness, recurrence, and poor clinical outcome, raising the possibility that its modification by PI(4,5)P2could also contribute to cancer-associated pathways (161). In HPV8 E6-expressing keratinocytes, the total cellular levels of CAND1 and SND1 remain unchanged, indicating that the increased PI(4,5)P2 signal associated with these proteins reflects enhanced PIPoylation rather than altered protein abundance (145). Together, these data support a model in which altered nuclear PI(4,5)P2 and protein-specific PIPoylation contribute to the transcriptional reprogramming observed in HPV-mediated carcinogenesis.

This concept aligns with the recognition that several enzymes regulating phosphoinositide turnover can shuttle between the cytoplasm and nucleus, thereby linking lipid signaling across these compartments and creating points at which disruptions in PIP homeostasis may contribute to cancer progression (162).

## Conclusion

Lipidation emerges as a central mechanism linking membrane organization with the dynamic behavior of BMCs across cellular compartments. Classical covalent lipidations, palmitoylation, myristoylation, and prenylation are established regulators of protein localization, stability, and signaling at membranes and play an important role in the membrane raft organization (21, 38). By influencing local lipid-protein interactions and hydrophobicity, these modifications determine how proteins partition between ordered and disordered membrane regions (21, 57, 58, 59, 60). In the cytoplasm, lipidation can anchor condensate-forming proteins to defined membrane domains, enabling coupling between protein phase separation and membrane-based processes such as lipid-domain formation and membrane remodeling (75, 76, 78). This ability to modulate condensate wetting and curvature extends the regulatory reach of lipidation beyond traditional signaling contexts. In autophagy, for example, LC3 phospholipidation provides a mechanistic bridge between phase-separated cargo condensates and membrane expansion, demonstrating that lipidation can both initiate and stabilize condensate-membrane coupling (94, 96, 99, 102).

Within the nucleus, the principles governing this lipidation-phase separation interplay persist but are adapted to a membrane-free environment. Recent studies reveal that elevated nuclear PI(4,5)P2 levels and phospholipidation influence protein function, chromatin organization, and transcriptional activity (111, 145). PI(4,5)P2, a well-characterized signaling lipid, functions here as an organizing factor that contributes to membrane-less compartmentalization through LLPS (134, 163, 164). Its interactions with IDRs of transcriptional regulators, chromatin remodelers, and RNA-binding proteins highlight how amphiphilic lipids can modulate condensate composition and material properties even in aqueous nuclear environments (122). The mechanisms underlying PI(4,5)P2 conjugation remain unresolved, but evidence supports both electrostatic and potential covalent modes of association regulating condensation, suggesting a continuum between reversible lipid-protein interactions and more stable forms of phospholipidation (120, 145, 148, 149, 150, 153, 165). This duality is consistent with models of amphiphile-stabilized nuclear microdomains and with recent observations of RNA-dependent PI(4,5)P2 clustering in nuclear condensates (9, 126, 130). From this perspective, the proposed concept of phosphoinositide lipidation represents a hypothesis that extends established energetic principles of lipid-protein interactions to membraneless nuclear environments. Beyond modulating phase behavior, phosphoinositides may also act as structural components of pre-assembled nuclear condensates, thereby shaping their biochemical environment and influencing their condensation dynamics. This concept redefines phosphoinositides not exclusively as diffusible signaling molecules, but also as pivotal regulators of nuclear architecture. While direct covalent attachment of phosphoinositides to nuclear proteins has not been definitively demonstrated, its verification would provide a mechanistic explanation for how lipid cues are integrated into chromatin regulation and transcriptional control.

Taken together, these findings suggest that lipidation-dependent processes follow a consistent regulatory pattern across cellular compartments. At membranes, lipidation defines spatial organization and nanodomain stability. In the cytoplasm, lipidation couples phase-separated assemblies with membrane remodeling. In the nucleus, phosphoinositides, such as PI(4,5)P2, extend lipid regulation to membrane-less systems. Recognizing lipidation and phospholipidation as determinants of LLPS thus integrates lipid metabolism with the molecular logic of condensate formation. This framework elucidates the mechanisms by which cells orchestrate biochemical organization across compartments and underscores the significance of lipid-dependent mechanisms as potential drivers of disease processes, including cancer and viral infection. A comprehensive understanding of these cross-compartmental lipidation pathways may ultimately reveal new molecular targets within the lipid-condensate interface that governs cellular structure and function.

## Data availability

No datasets were generated or analyzed during the current study.

## Conflicts of interest

The authors declare that they do not have any conflicts of interest with the content of this article.
